# Editorial: Current advances in the understanding and management of pelvic organ prolapse

**DOI:** 10.3389/fsurg.2025.1765723

**Published:** 2026-01-07

**Authors:** Carlo Ronsini

**Affiliations:** Unit of Gynecologic Oncology, National Cancer Institute, IRCCS, Fondazione “G. Pascale”, Naples, Italy

**Keywords:** biomechanics of pelvic support, epidemiology and pathophysiology, laparoscopic, minimally invasive surgery, molecular mechanics, pelvic floor dysfunction, pelvic organ prolapse, robotic interventions

Pelvic organ prolapse (POP) remains one of the most common and burdensome pelvic floor disorders worldwide, affecting millions of women and significantly impairing their quality of life. Despite extensive clinical experience and ongoing refinements in surgical techniques, the underlying pathophysiology and optimal approaches for prevention and management continue to evolve. The Research Topic “*Current Advances in the Understanding and Management of Pelvic Organ Prolapse”* gathers ten diverse contributions that collectively enhance our knowledge of POP, from molecular mechanisms to public health implications, obstetric factors, and advances in surgical treatment.

A major step forward in elucidating the biological foundations of POP is offered by the transcriptomic analysis performed by Rui et al. By profiling coding and non-coding RNAs in uterosacral ligament tissue of postmenopausal women, the authors highlight extensive dysregulation of pathways involved in extracellular matrix remodeling, inflammation, cytoskeletal organization, and cellular senescence. Among their findings, downregulation of the *lncRNA FLJ20021* and its association with altered collagen and MMP expression underscore how cellular and molecular alterations can translate into structural weakening of pelvic support. These results bring new clarity to the biological processes that predispose to prolapse and open potential avenues for biomarker discovery and targeted therapies. Tian et al. offer a complementary perspective by proposing a new biomechanical framework for understanding POP. Moving beyond static descriptions of pelvic support defects, the authors view POP as a disorder of dynamic mechanical balance involving the coordinated action of the levator plate, the perineal body, the posterior vaginal fornix, and neuromuscular control. Their model explains common patterns of organ descent and recurrence after surgery, suggesting that long-term success may depend not only on anatomical restoration but also on reestablishing balanced force transmission throughout the pelvic floor. Together, the molecular and biomechanical factors show POP as a complex interaction between tissue degeneration and altered mechanical loading.

The broader public health implications of pelvic floor disorders are highlighted in two population-based studies. Wang et al., using Global Burden of Disease data from 1990 to 2021, show that although age-standardized incidence and disability-adjusted life-year rates have declined slightly, the total number of women living with POP has increased substantially, particularly in low-SDI regions. This gap between population aging and healthcare capacity underscores the need for policies that expand access to high-quality pelvic floor care across socioeconomic groups. At the national level, Chen et al. examine more than 1,600 Chinese women with untreated pelvic floor dysfunction and illustrate how symptoms like urinary incontinence, POP, pelvic pain, and fecal incontinence significantly impact physical and mental well-being over time. Their findings emphasize the importance of early recognition and intervention to mitigate long-term deterioration and reduce the societal impact of these conditions.

One of the most common assaults on the pelvic floor is undoubtedly childbirth, and the systematic review by Chen et al. offers essential insights into the role of labor characteristics. Their meta-analysis of randomized controlled trials shows that prolonging the second stage of labor in first-time mothers is associated with higher rates of urinary incontinence, pelvic pain, and urinary retention and may increase the risk of later POP. These findings highlight the importance of obstetric practices that focus not only on immediate neonatal outcomes but also on long-term maternal pelvic floor health.

## Our special issue focused not only on the etiology, but also on the treatment of POP

Several contributions in this Research Topic emphasize surgical management, highlighting the importance of operative intervention for many women with symptomatic prolapse. Innovations in minimally invasive techniques, especially sacrocolpopexy, are prominently featured. Ota et al. compare a newly developed vaginal manipulator with the traditional spatula during robotic sacrocolpopexy and find that the new device improves apical exposure, reduces hospital stays, and enhances postoperative support while maintaining safety and procedural efficiency. However, the need for constant innovation should not always be accepted blindly, as demonstrated by the critical eye presented by Jamaleddine et al., which critically analyze the literature on robotic sacrocolpopexy, discussing its benefits, including greater surgical dexterity, better visualization, and less blood loss, while also addressing challenges such as operative time, cost, and the lack of standardized techniques across centers. Expanding on these observations, Strauss et al. compare laparoscopic and robotic-assisted sacrocolpopexy in relation to patient characteristics and demonstrate that robotic assistance can attenuate the negative impact of advanced age, higher BMI, and increased parity on operative duration and postoperative recovery, suggesting that robotics may provide particular benefit in anatomically complex or high-risk patients. Surgical decision-making also entails careful consideration of potential complications. A multicenter retrospective study led by my group (Ronsini et al.) examines whether the addition of cystopexy during laparoscopic hysterectomy for uterine prolapse improves outcomes. Their findings show a significantly higher rate of postoperative complications when cystopexy is performed, without a corresponding reduction in prolapse recurrence, suggesting that additional procedures should be incorporated selectively rather than routinely. This work follows on from previous research conducted by our group, which demonstrated that, during laparoscopic hysterectomy for POP, certain ancillary procedures such as Shull's colposuspension to the uterosacral ligaments can provide good control of pelvic statics without the need for additional surgical procedures, which would prolong the operation and expose patients to potential comorbidities (1). Attention to individual surgical steps remains crucial for optimising postoperative outcomes, as also demonstrated by the study by Lu et al. (2) on hidden blood loss in laparoscopic myomectomy. This study provides methodological insights and demonstrates that unrecognized intraoperative bleeding may affect postoperative anemia and recovery, underscoring the importance of precise perioperative management in all minimally invasive gynecologic procedures. Minimally invasive surgery plays a central role in POP, and the sophistication of surgical techniques presents ever-increasing challenges. This compels us, as experts in the field, to critically examine the role of training and the learning pathways required to master this type of surgery. It has previously been demonstrated that a threshold of 20 procedures is the minimum number needed for a surgeon to be considered “adequate” for the minimally invasive treatment of POP (3).

In conclusion, these contributions present a coherent picture. POP is not merely an anatomical failure but results from interconnected biological, mechanical, obstetric, and demographic factors. Advances in molecular profiling and biomechanical modeling deepen our understanding of pathogenesis, while epidemiologic and public health research highlight the urgency of improving access to care. Simultaneously, innovations in surgical techniques, especially robotic and laparoscopic methods, are enhancing our ability to restore function with precision and durability, though cost, standardization, and patient selection remain essential considerations.

Taken together, the studies included in this Research Topic advance the field toward a comprehensive, evidence-based approach for managing pelvic organ prolapse. Ongoing interdisciplinary research, along with careful application in clinical practice and public health strategies, will be crucial to reducing the burden of POP and enhancing outcomes for women worldwide.

## References

[1] Restaino S Ronsini C Finelli A Santarelli A Scambia G Fanfani F. Laparoscopic approach for Shull repair of pelvic floor defects. J Minim Invasive Gynecol. (2018) 25(6):954. 10.1016/j.jmig.2017.12.016 29289624

[2] Ye M Zhou J Chen J Yan L Zhu X. Analysis of hidden blood loss and its influential factors in myomectomy. J Int Med Res. (2020) 48(5):300060520920417. 10.1177/0300060520920417 32397777 PMC7223209

[3] Cianci S Ronsini C Riemma G Palmara V Romeo P La Verde M Multicentric data analysis of the learning curve for laparoscopic Shull’s repair of pelvic floor defects. Front Surg. (2024) 11:1396438. 10.3389/fsurg.2024.1396438 38948480 PMC11212461

